# Cognitive training can reduce the rate of cognitive aging: a neuroimaging cohort study

**DOI:** 10.1186/s12877-016-0194-5

**Published:** 2016-01-14

**Authors:** Ting Li, Ye Yao, Yan Cheng, Bing Xu, Xinyi Cao, David Waxman, Wei Feng, Yuan Shen, Qingwei Li, Jijun Wang, Wenyuan Wu, Chunbo Li, Jianfeng Feng

**Affiliations:** 1 Shanghai Key Laboratory of Psychotic Disorders, Shanghai Mental Health Center, Shanghai Jiao Tong University School of Medicine, Shanghai, China; 2 Centre for Computational Systems Biology, Fudan University, Shanghai, China; 3 Department of Psychiatry, Tongji Hospital of Tongji University, Shanghai, China; 4 Department of Psychiatry, Tenth People’s Hospital of Tongji University, Shanghai, China; 5 Shanghai Center for Mathematical Sciences, Fudan University, Shanghai, China; 6 Department of Computer Science, University of Warwick, Coventry, CV4 7AL UK

**Keywords:** Aging, Cognitive training, Intrinsic brain activity, Functional magnetic resonance imaging, Functional connectivity, Entropy

## Abstract

**Background:**

The neural mechanisms underlying the restorative effects of cognitive training on aging brains remain unclear. To address this issue, we examined the relationship between changes in spontaneous brain activity and cognitive performance that occur after cognitive training.

**Methods:**

Participants were older adults who were part of a randomized control trial within a larger longitudinal cognitive training study. We conducted single-domain and multi-domain cognitive training in two respective intervention groups. Participants were trained for 1 h, twice a week, for 12 weeks. Cognition was assessed in all participants and magnetic resonance images were obtained at baseline and 1 year after training. To assess spontaneous fluctuations in brain activity, we acquired resting-state fMRI data. Two indices—functional entropy and time-domain entropy—were used to measure the effects of training. Functional entropy increases with aging, and indicates disruptions in functional conectivity. Time-domain entropy decreases with aging, and indicates structural alterations in the brain and blood-flow reduction.

**Results:**

Seventy participants completed the study: 26 in the multi-domain cognitive training group (70.38 ± 3.30 yrs), 27 in single-domain group (70.48 ± 3.93 yrs), and 17 in a control group (68.59 ± 3.24 yrs). Functional entropy increased significantly less in the multi-domain (*p* = 0.047) and single-domain groups (*p* = 9.51 × 10^−4^) compared with the control group. In the multi-domain group, this was true in the paracentral lobule (*p =* 0.004, Bonferroni corrected *p* < 0.05). Time-domain entropy also improved with training. Compared with controls, time-domain entropy in the multi-domain group decreased less in the inferior frontal gyrus pars opercularis (*p =* 3.59 × 10^−4^), the medial part of superior frontal gyrus (*p =* 1.17 × 10^−5^), and the thalamus (*p =* 4.72 × 10^−5^), while that in the single-domain group decreased less in the cuneus (*p =* 2.58 × 10^−4^, Bonferroni corrected *p* < 0.05). Additionally, changes in regional entropy for some regions such as hippocampus significantly correlated with improvements in cognitive performance.

**Conclusions:**

Cognitive training can induce plastic changes in neural functional connectivity of healthy older people, and these changes may underlie the positive effect of cognitive training.

**Trial registration:**

ChiCTR-TRC-08000732 (Date of registration: 5th November, 2008).

**Electronic supplementary material:**

The online version of this article (doi:10.1186/s12877-016-0194-5) contains supplementary material, which is available to authorized users.

## Background

Normal aging of the brain is associated with memory loss and cognitive impairments that can interfere with daily routines [1] and affect the capacity of older people to live independently. Additionally, cognitive impairment is associated with a higher mortality risk [2]. Therefore, researching how to prevent cognitive impairment with aging is essential, especially given the current aging crisis that is facing the world [3]. In addition to pharmacological therapies, the past few decades have seen a rise in research aimed at non-pharmacological intervention, such as cognitive training (CogTr) and physical exercise [4–6]. Increasing mental activity in older people by directed CogTr interventions is a particularly promising approach for delaying age-related cognitive decline [7]. Results from randomized controlled trials suggest that cognitive training can even induce improvements in cognition [8–11].

However, the neural mechanisms underlying the restorative effects of CogTr on aging brains remains unclear and are subject to debate [12, 13]. The essential difficulty, as pointed out in Owen et al. [12], lies in the difficulty of finding any evidence that the effects of CogTr transfer to untrained tasks. One promising way to fully assess the impact of CogTr on aging individuals and to explore its mechanisms, is to studying cortical changes (neural plasticity) via neuroimaging techniques such as functional magnetic resonance imaging (fMRI) [14, 15]. fMRI is a noninvasive imaging method that assesses activity in regions of the brain based on the blood oxygen level-dependent (BOLD) contrast, and cognitive neuroscience research has already revealed brain plasticity throughout life that means changes and reorganization occurring even in the aging brain [16]. Cognitive training-related plastic changes in the brain have been reported in several task-state fMRI studies. For instance, activity in a number of frontal and parietal regions involved in memory, as well as activity in the bilateral hippocampus, was found to be greater after memory training in both healthy older people and those with mild cognitive impairment [15]. Further, semantic encoding-strategy training increased brain activity in the medial superior frontal gyrus, right precentral gyrus, and left caudate of older adults during intentional encoding [17].

Recently, using resting-state fMRI to complement traditional task-state fMRI is becoming more common. With resting-state fMRI, brain regions co-activated while not engaged in any specific task can be identified using spontaneous fluctuations in brain activity. Evidence increasingly suggests that temporal fluctuations in resting-state fMRI, in particular low frequency components (<0.1Hz), arise primarily from spontaneous fluctuations in brain metabolism and intrinsic neuronal activity [18, 19]. Researchers agree that spontaneous neuronal activity plays a fundamental role in brain function, maintaining brain integrity and its capacity to deal effectively with future demands [20, 21].

Exploring the effects of cognitive training on spontaneous brain activity in older adults may provide fundamental proof for the neural mechanism underlying the effects of cognitive training. Therefore, we conducted a neuroimaging investigation as a sub-study of the longitudinal CogTr program [11]. In the current study, we used two entropy indices, time-domain entropy and functional entropy, to quantify spontaneous neuronal activity. Entropy is a fundamental concept originating in statistical physics and information theory, and can be used to measure the degree of uncertainty within a probability distribution [22], with higher entropy reflecting greater uncertainty [23].

Time-domain entropy was used to characterize the uncertainty of individual time series of spontaneous brain activity that may indirectly reflect the complexity of brain structure. Aging has been found to be associated with a reduction in the time-domain entropy of spontaneous brain activity [24–26]. Additionally, this measure of entropy was positively correlated with cognitive performance in the older people [25, 26]. A decreasing time-domain entropy with age (see Additional file 1 Section 3 for more details) reflects the lower oscillating frequency in BOLD signals as we get older, which can result from the loss of cortical and subcortical connections, loss of white matter or demyelination [27, 28], synaptic degeneration [29, 30], neurochemical alteration [31], or reduced blood flow [32]. In addition to time-domain entropy, the asynchrony of the time series among brain regions can be characterized by functional entropy, which can be viewed as an alternative expression of functional connectivity. Functional entropy measures the relative orderliness of the time series patterns among brain regions, with lower functional entropy values denoting greater conditional regularity or synchronicity. Yao et al. found that in a population of healthy individuals, functional entropy naturally increased with age, as evidenced by correlations in spontaneous brain activity that became more widely distributed [33], indicating age-related functional conectivity disruption. The first aim of our study was to determine if these two entropy indices change as a result of cognitive training.

Reduced lateralization of brain activity is another important feature observed in aging populations. This is known as “hemispheric asymmetry reduction in older adults” (HAROLD) [34]. According to this model, aging brains recruit additional brain regions to face task demands. This may reflect age-related dedifferentiation processes or compensatory functional brain responses [35, 36]. The second aim of our study was to explore the effect of cognitive training on age-related HAROLD.

Our hypothesis can be divided into three parts: 1) Cognitive training leads to changes in the entropy of spontaneous neural activity in healthy older adults, including reductions in the extent to which time-domain entropy decreases and in how much functional entropy increases. 2) Cognitive training reduces HAROLD. 3) Brain changes induced by cognitive training have correlations with cognitive performance. Because the effects of different cognitive training methods remain unknown, we investigated the effects of multi-domain and single-domain cognitive training.

## Methods

The study was approved by the Human Research Ethics Board of Tongji Hospital in Shanghai, China. Written informed consent was obtained from all participants before the experiment (LL (H)-09-04).

### Participants

**Fig. 1:**
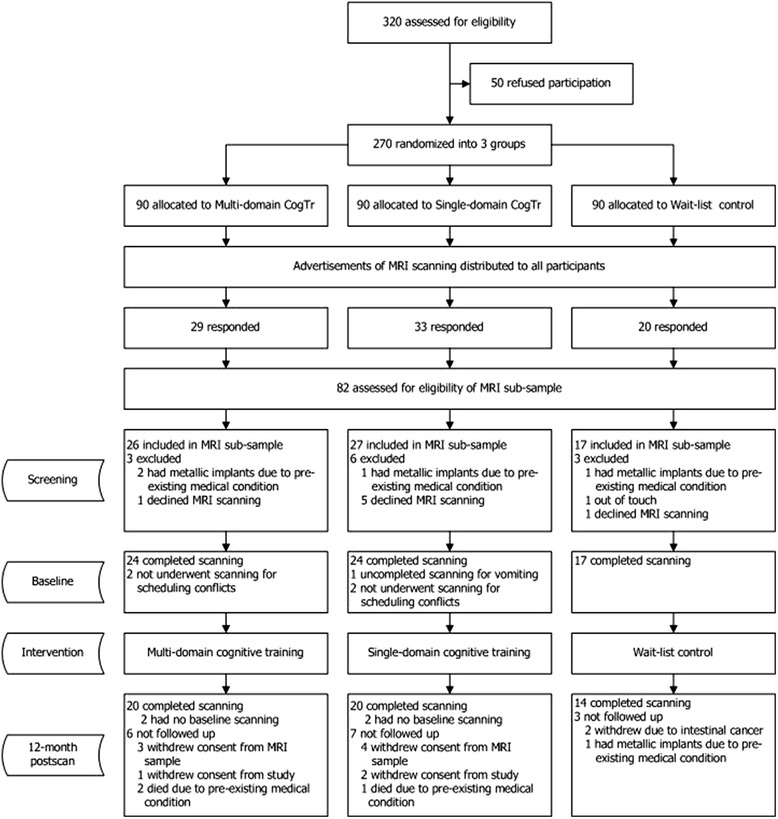
Flow of participants through the trial. ‘Refused’ or ‘Declined’ due to lack of interest in continuing, opposition from other family member, MRI examination in hospital recently, physical discomfort. ‘Died’ includes cancer, stroke, and cardiac sudden death

Participants were from a longitudinal CogTr study. The longitudinal CogTr study was a randomized control trial in healthy older adults, using three groups, including two intervention groups (single-domain CogTr and multi-domain CogTr training) and one wait-list control group [11]. Details can be seen in Fig. 1.

Participants were healthy older adults with normal functional capacity, living independently in the community. They were recruited between March 2008 and April 2008 from three community centers around Tongji Hospital through a notice/broadcast dispatched by the local community service institute. Eligibility criteria included: age 65–75 years; education ≥ 1 year; no difficulties with hearing, vision, or communication; no severe physical disease or psychotic disorder; and no obvious cognitive decline (a score on the Chinese version of the Mini-Mental State Examination ≥ 19 [37]). Exclusion criteria included: obvious cognitive decline such as AD; history or clinical evidence of neurological disease or psychiatric disorder such as brain cancer, major depressive disorder, or schizophrenia. Because the training-related changes predicted in our study have no relation with handedness [38], we enrolled both right- and left-handed participants. One of the 26 participants in the multi-domain training group was left-handed, as was one of the 27 in the single-domain training group.

### Study design

We used a controlled and assessor-blind design to test the effect of CogTr on spontaneous brain activity and cognitive capacity. Magnetic resonance imaging (MRI) was performed for all participants at baseline and at 1 year after training ending.

### Interventions

We conducted single-domain or multi-domain CogTr in the two respective intervention groups. Participants completed 1 h of training, twice a week, for 12 weeks. The training procedure was administered in a classroom at Tongji Hospital. The multi-domain training targeted memory, reasoning, problem-solving strategies, visuospatial map-reading skills, and making handcraft. The single-domain CogTr focused specifically on reasoning, including the “towers of Hanoi” puzzle, numerical reasoning, Raven’s progressive matrices, and verbal reasoning. During the first 15 min of every training session, the trainer gave a lecture about a common disease in aging people such as hypertension. Then, the participants were taught about a certain cognitive strategy or technique via a slide presentation for the next 30 min. The training tasks were administered mainly via pen/paper. The newly learned skill was reinforced by solving a few real-life problems (for more details, see our previous study [10]). The control group did not undergo training and was used as a match for the social contact associated with CogTr. All participants including the control group were given a lecture about healthy living every 2 months.

### Measures

#### Cognitive testing

All participants were, initially, given a cognitive capacity assessment, and this was taken as the baseline for any subsequent changes. One year after intervention another cognitive capacity assessment was conducted. The measures included the Repeatable Battery for the Assessment of Neuropsychological Status [39] (RBANS, Form A), which has been shown to have good reliability and validity in a sample of Chinese community-living older people [40], the Color Word Stroop test (CWST) [41], the Visual Reasoning test [42] and the Trail Making test (TMT) [43].

#### MRI acquisition

Scanning was performed using a Siemens 3.0 Tesla Allegra scanner (Erlangen, Germany) at East China Normal University, Shanghai, China. Foam padding was used to minimize head motion for all subjects. Functional images were acquired using a single-shot, gradient-recalled echo planar imaging sequence (repetition time = 2000 ms, echo time = 25 ms and flip angle = 90°). Thirty-two transverse slices (field of view = 240 × 240 mm^2^, in-plane matrix = 64 × 64, slice thickness = 5 mm, voxel size = 3.75 × 3.75 × 5 mm^3^), aligned along the anterior commissure-posterior commissure line were acquired. For each subject, a total of 155 volumes were acquired, resulting in a total scan time of 310 s. Subjects were instructed simply to rest with their eyes closed, not to think of anything in particular, and not to fall asleep. Subsequently, high-resolution T1-weighted anatomical images were acquired in the sagittal orientation using a magnetization-prepared rapid gradient-echo sequence (repetition time = 1900 ms, echo time = 3.43 ms, flip angle = 9°, field of view = 256 × 256 mm^2^, matrix size = 256 × 256, slice thickness = 1 mm, voxel size = 0.9375 × 0.9375 × 1 mm^3^ and 160 slices) on each subject.

#### Data preprocessing

The first 10 volumes of the samples were discarded to allow for scanner stabilization and subject adaptation to the environment. Preprocessing of fMRI data was then conducted using Statistical Parametric Mapping 8 (SPM8) (http://www.fil.ion.ucl.ac.uk/spm), Resting-State fMRI Data Analysis Toolkit (REST V1.8) (http://restfmri.net/forum/), and Data Processing Assistant for Resting-State fMRI (DPARSF V2.2) (http://restfmri.net/forum/dparsf_v2_2). The remaining functional scans were first corrected for within-scan acquisition-time differences between slices, and then realigned to the middle volume to correct for inter-scan head motions. Subsequently, the functional scans were spatially normalized to a standard template [44] (Montreal Neurological Institute) and resampled to 3 × 3 × 3 mm^3^. Data was then smoothed, and after normalization and smoothing, BOLD signals of each voxel were first detrended to remove any linear trend, and then passed through a band-pass filter (0.01 ~ 0.08 Hz) to reduce low-frequency drift and high-frequency physiological noise. Finally, nuisance covariates including head motions, global mean signals, white matter signals, and cerebrospinal signals were regressed out from the BOLD signals. After data preprocessing, the time series were extracted in each region of interest (ROI) by averaging the signals of all voxels within that region and then linearly regressing out the influence of head motion and global signals. In our present study, the automated anatomical labeling atlas [45] (AAL) was used to divide the brain into 90 regions of interest (ROIs) (45 per hemisphere). The names of the ROIs and their corresponding abbreviations are listed in Tao’s work [46].

#### Method for calculating entropies

After data preprocessing, time-domain data was extracted for each ROI by averaging the signals of all voxels within the region. We then calculated the Approximate Entropy [47] of the time series for each brain region with the following key parameters: *window length*, *m* = 1 time point; *regularity* of the time series, *r =* 0.12 × standard deviations. We termed the Approximate Entropy of each individual brain region the *regional time-domain entropy*. We proceeded to determine the average of all the regional time-domain entropies, and called this the *time-domain entropy*, following the rationale that different regional time-domain entropies could be considered as different realizations of the time-domain entropy.

We next calculated Pearson correlation coefficients for each pair of brain regions. The total number of correlations was 4005 because this is the number of functional links available from the AAL template. With these data, we constructed a whole-brain functional network. The 4005 different correlation coefficients require an indicator to represent features of the whole-brain functional network. We considered two possible approaches.

1) In the first approach, we took the values of the correlation coefficients as a realization of a random variable, which lies in the range [−1, 1]. We then assumed the brain entropy was the Shannon entropy [48]. Thus, we sorted all 4005 correlation coefficients into 20 class intervals of equal width, and determined the frequency (*π*) of each class. These frequencies were used to calculate the Shannon entropy for the whole brain, namely the sum of *-π*log (*π*). This can be considered the entropy of a discrete distribution, and following past usage [33], we called this result the *functional entropy*.

2) The alternative approach was to calculate the entropy for every single brain region. This method considers the functional links between a selected region and the other 89 regions. Thus, we could extract 89 Pearson correlation coefficients for each region by comparing its BOLD time series with those of the other 89 regions. We also separated these into 20 equal-width class intervals, and calculated the Shannon entropy of each region. We called the entropy of each brain region the *regional functional entropy*.

#### Statistical inference

In this paper, we used several statistical methods. The first and most basic one was the Pearson product-moment correlation coefficient. This was used to describe the relationship between each pair of brain regions and allowed us to quantify the relationship between the two types of entropy and other relevant quantities. Additionally, we used the Approximate Entropy [49] to extract the entropy of the time series. We used a Student's *t*-distribution to calculate the *p* value of the Pearson correlation coefficient, and used linear regression to remove the effects of age, gender, and education. We used the t-tests to compare measurements between each intervention group and control group. Additionally, a Chi-squared test was employed to test the comparability of gender, and an F test was utilized to test the comparability of age and education between the different groups.

We also collected cognitive behavioral data using the RBANS, CWST, visual reasoning tests, and TMT scores, and used before and after (1 year later) differences in these measures to calculate changes in cognitive ability. We used linear regression to determine the relationship between behavioral score changes and age, gender, and education in the control participants. We then took the control scores as a baseline and calculated the theoretical score-change in the multi-domain and single-domain training groups based on their age, gender, and education. We then compared the difference between the predictions and the actual values, and considered any difference to be an effect of the CogTr. We applied the same method when comparing the entropy values.

It should be emphasized that our results are primarily based on entropy—an informational concept—at the time and functional connectivity levels. To have a direct comparison with more conventional approaches, we also performed functional connectivity analysis and provide details in Additional file 1 section 8.

## Results

### Demographic information

Eighty-two participants responded and were assessed for baseline MRI scanning eligibility. Among them, 70 were eventually included in the MRI subsample. They were divided into three groups based on type of cognitive training: multi-domain (*n* = 26, mean age 70.38 ± 3.30 yrs), single-domain (*n* = 27, mean age 70.48 ± 3.93 yrs), and control (no training, *n* = 17, mean age 68.59 ± 3.24 yrs). Sixty-five participants completed the baseline MRI scanning. No significant differences in demographics were found between the participants who responded to advertisements and included in the imaging subsample. Fifty-four participants completed the 1-year follow-up. The dropout rate during the study was 28.57 %. The flow of participants through the entire study is illustrated in Fig. 1. The intervention groups were comparable with the control group with regard to age, gender, and education at each assessment. No significant differences in age, education, or gender were found across the three groups (*p* > 0.05). Subject characteristics are summarized in Table 1.

**Table 1:** Characteristics of participants at different time points in the study

		Multi-domain CogTr group	Single-domain CogTr group	Control group	*F/ χ* ^2^	*p*
Age ($\overline{\mathrm{x}}$ ± SD)	Baseline	70.54 ± 3.23	69.96 ± 3.85	68.59 ± 3.24	1.604	0.209
	1 year after training	72.28 ± 3.46	71.61 ± 3.97	70.93 ± 3.63	0.527	0.594
Education ($\overline{\mathrm{x}}$ ± SD)	Baseline	10.58 ± 4.14	9.33 ± 3.74	11. 00 ± 3.85	1.051	0.356
	1 year after training	11.67 ± 3.20	9.39 ± 4.22	10.71 ± 3.34	1.78	0.180
Gender (male: female)	Baseline	17:7	13:11	9:8	1.867	0.39
	1 year after training	13:5	8:10	9:5	3.042	0.218

### Cognitive capacity

RBANS test score significantly improved (*t =* 2.75, *p =* 0.007) in the multi-domain CogTr group. Specifically, it was especially improved in language (*t =* 2.13, *p =* 0.024), attention (*t =* 1.95, *p =* 0.034) and delayed memory (*t =* 2.74, *p =* 0.007). In contrast, the single-domain CogTr group showed no significant changes. Moreover, CWST and TMT scores did not show any significant improvement in either group. Lastly, the visual reasoning score improved significantly in the single-domain CogTr group (*t =* 2.32, *p =* 0.017), and trended toward improvement in the multi-domain CogTr group (*t =* 1.61, *p =* 0.063).

### Spontaneous brain activity

**Fig. 2:**
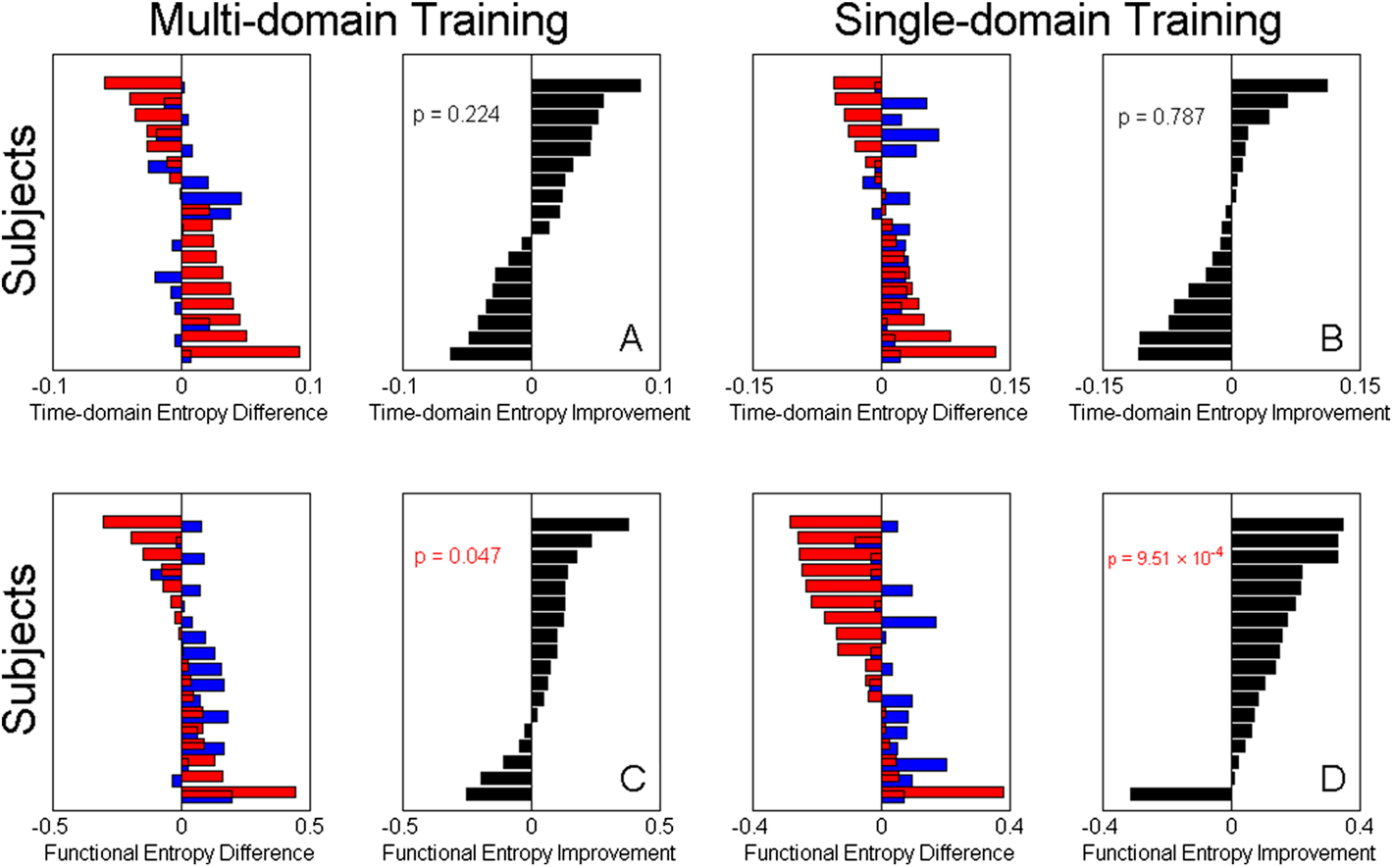
Entropy change in cognitive training groups. The entropy differences (black bars) between the predictions (*blue bars*) and actual values (red bars) indicated the CogTr effect on the entropy measures. Panels (**a**) & (**b**): The mean decrease in time-domain entropy was found to be smaller in both multi-domain CogTr group (*t = −*0.77, *p =* 0.224) and single-domain CogTr group (*t =* 0.81, *p =* 0.787) when compared with the prediction for people with no training. Panels (**c**) & (**d**): The mean increase in functional entropy was significantly reduced in both multi-domain CogTr (*t =* 1.77, *p =* 0.047) and single-domain CogTr (*t =* 3.67, *p =* 9.51 × 10^−4^) groups. Subjects are ranked according to the entropy values

We found a number of differences between groups that were associated with spontaneous brain activity. The mean increase in functional entropy was significantly reduced in both multi-domain CogTr (*t =* 1.77, *p =* 0.047) and single-domain CogTr (*t =* 3.67, *p =* 9.51 × 10^−4^) groups. Additionally, the mean decrease in time-domain entropy was found to be smaller in both multi-domain (*t = −*0.77, *p =* 0.224) and single-domain CogTr group (*t =* 0.81, *p =* 0.787) when compared with the prediction for people with no training, as shown in Fig. 2.

**Fig. 3:**
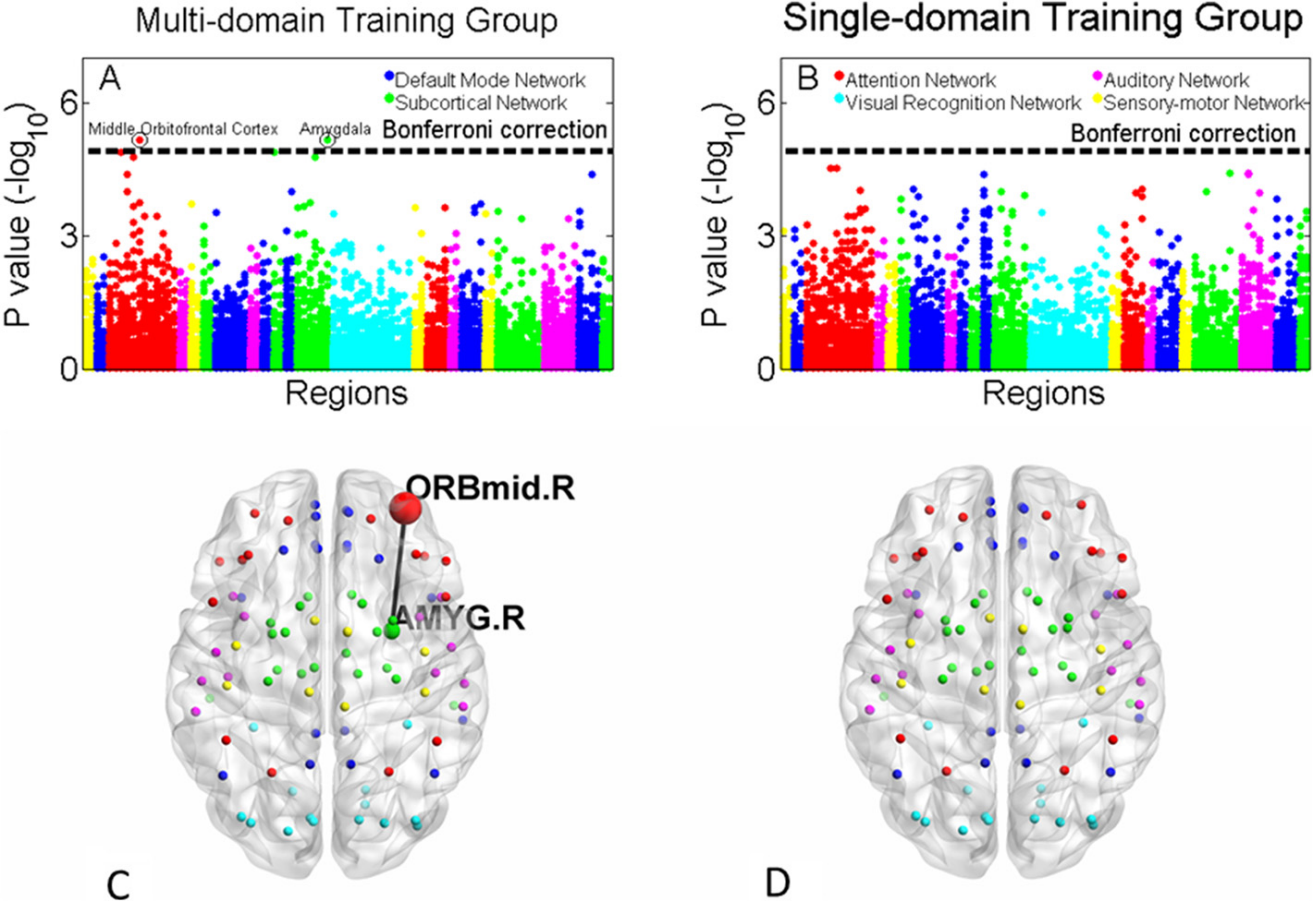
Functional connectivity change in training groups. Panels (**a**) & (**c**) In the Multi-domain training group, time-domain entropy in the amygdala (right hemisphere) correlated significantly with that in the middle orbitofrontal cortex (right hemisphere) (*p* = 7.2 × 10^−6^, Bonferroni corrected *p* < 0.05). Panels (**b**) & (**d**) Time-domain entropy did not correlate between any pairs of regions in the single-domain training group

All regional time-domain entropies significantly decreased with age, as shown in Section 5 of the Additional file 1. At the regional level, time-domain entropy decreased significantly less in several brain regions of those in the multi-domain group compared with controls. In particular, this was true in the inferior frontal gyrus pars opercularis (*t =* 4.12, *p =* 3.59 × 10^−4^, Bonferroni corrected *p* < 0.05), the medial part of superior frontal gyrus (*t =* 5.75, *p =* 1.17 × 10^−5^), and the thalamus (*t =* 5.07, *p =* 4.72 × 10^−5^). Similarly, time-domain entropy decreased significantly less the cuneus of those in the single-domain training group compared with controls (*t =* 4.27, *p =* 2.58 × 10^−4^, Bonferroni corrected *p* < 0.05). In the Multi-domain training group, time-domain entropy in the amygdala (right hemisphere) correlated significantly with that in the middle orbitofrontal cortex (right hemisphere) (*p* = 7.2 × 10^−6^, Bonferroni corrected *p* < 0.05). However, time-domain entropy did not correlate between any pairs of regions in the single-domain training group (Fig. 3).

As shown in Yao et al’s work [33], regional functional entropies in the paracentral lobule, hippocampus, parahippocampal gyrus, olfactory cortex, and middle frontal gyrus, increase the most with age. In our experiment, functional entropy in the paracentral lobule increased less in the multi-domain training group than in controls (*t =* 3.03, *p =* 0.004, Bonferroni corrected *p* < 0.05).

**Fig. 4:**
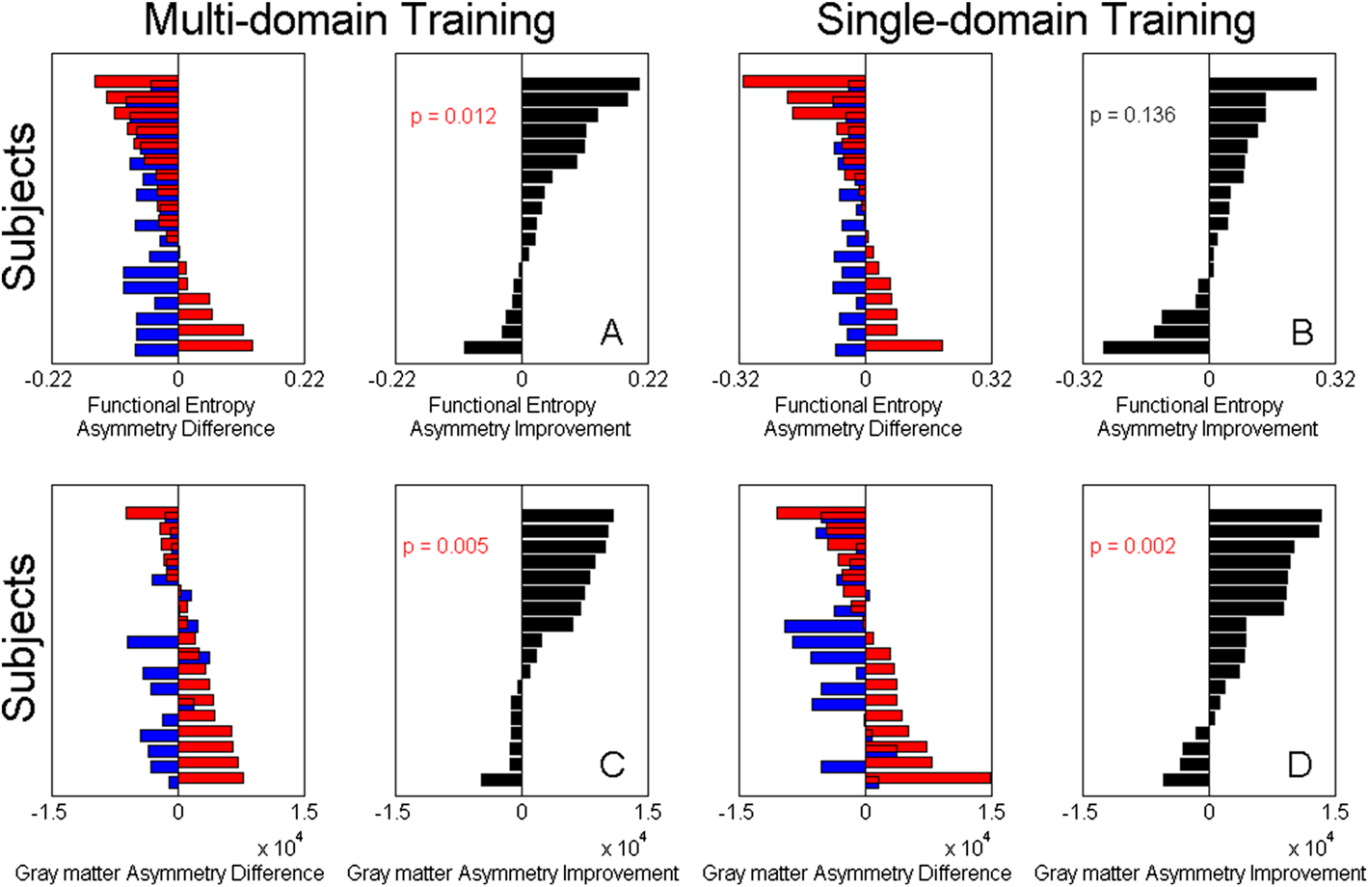
Asymmetry in functional entropy change in training groups. Panel (**a**): The functional entropy asymmetry differences (*black bars*) between predictions (*blue bars*) with actual values (*red bars*) indicated that asymmetry in functional entropy decreased significantly less in the multi-domain CogTr group than in controls (*t* = 2.48, *p =* 0.012). Panel (**b**): In contrast, it did not decrease less in the single-domain group (*t =* 1.13, *p =* 0.136). Panels (**c**) and (**d**): asymmetry in gray matter decreased significantly less in the multi-domain group (*t* = 2.93, *p* = 0.005) and single-domain group (*t* = 3.33, *p* = 0.002). Subjects are ranked according to the values

Differences in functional entropy between left and right hemispheres decreased with age, as shown in Section 6 of the Additional file 1. In particular, asymmetry in functional entropy decreased significantly less in the multi-domain CogTr group than in controls (*t* = 2.48, *p =* 0.012). In contrast, it did not decrease less in the single-domain training group (*t =* 1.13, *p =* 0.136) (Fig. 4a and b). Additionally, asymmetry in gray matter decreased significantly less in the multi-domain training group (*t* = 2.93, *p* = 0.005; Fig. 4c) and single-domain training group (*t* = 3.33, *p* = 0.002; Fig. 4d). Details are shown in Additional file 1, Section 4.

**Fig. 5:**
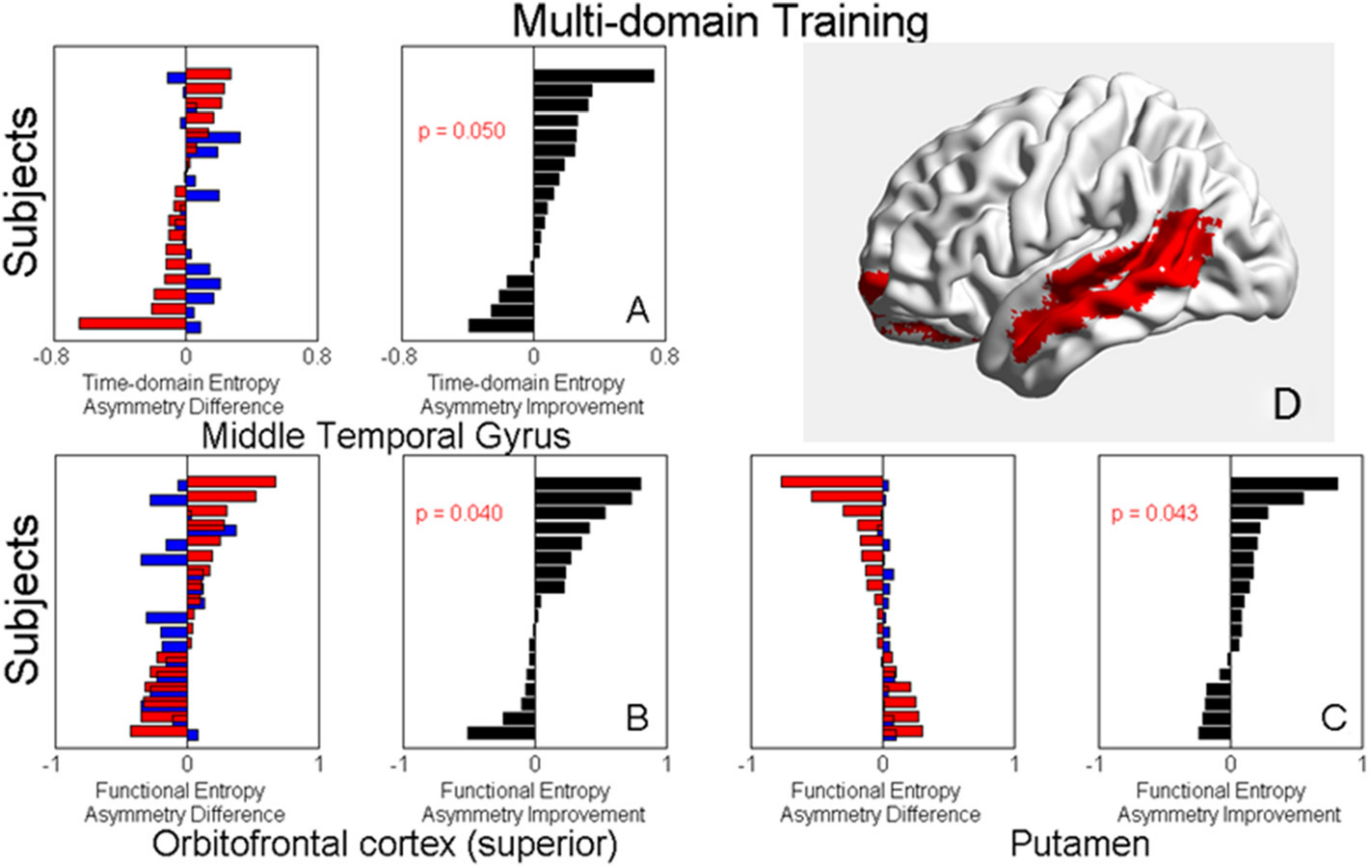
Entropy asymmetry change in multi-domain CogTr group. Panels (**a**), (**b**) and (**c**): Compared to controls, the amount of entropy asymmetry was significantly reduced in the middle temporal gyrus (*t =* 1.72, *p =* 0.050). **a** the orbital part of the superior frontal gyrus (*t =* 1.86, *p =* 0.040) (**b**) and the putamen (*t =* 1.82, *p =* 0.043). **c** of the multi-domain CogTr group. Panel (**d**): These regions found in (**a**), (**b**) and (**c**) are represented. Subjects are ranked according to the entropy values

Time-domain entropy in the middle temporal gyrus significantly increased with age, functional entropy significantly decreased with age in the orbital part of the superior frontal gyrus, and significantly increased with age in the putamen (Additional file 1, Section 7). Additionally, as shown in Fig. 5, compared to controls, the amount of entropy asymmetry was significantly reduced in the middle temporal gyrus (*t =* 1.72, *p =* 0.050), the orbital part of the superior frontal gyrus (*t =* 1.86, *p =* 0.040), and the putamen (*t =* 1.82, *p =* 0.043) of the multi-domain CogTr group. However, there was no similar significant reduction in the single-domain CogTr group.

### The correlation between cognitive capacity and resting state fMRI

**Fig. 6:**
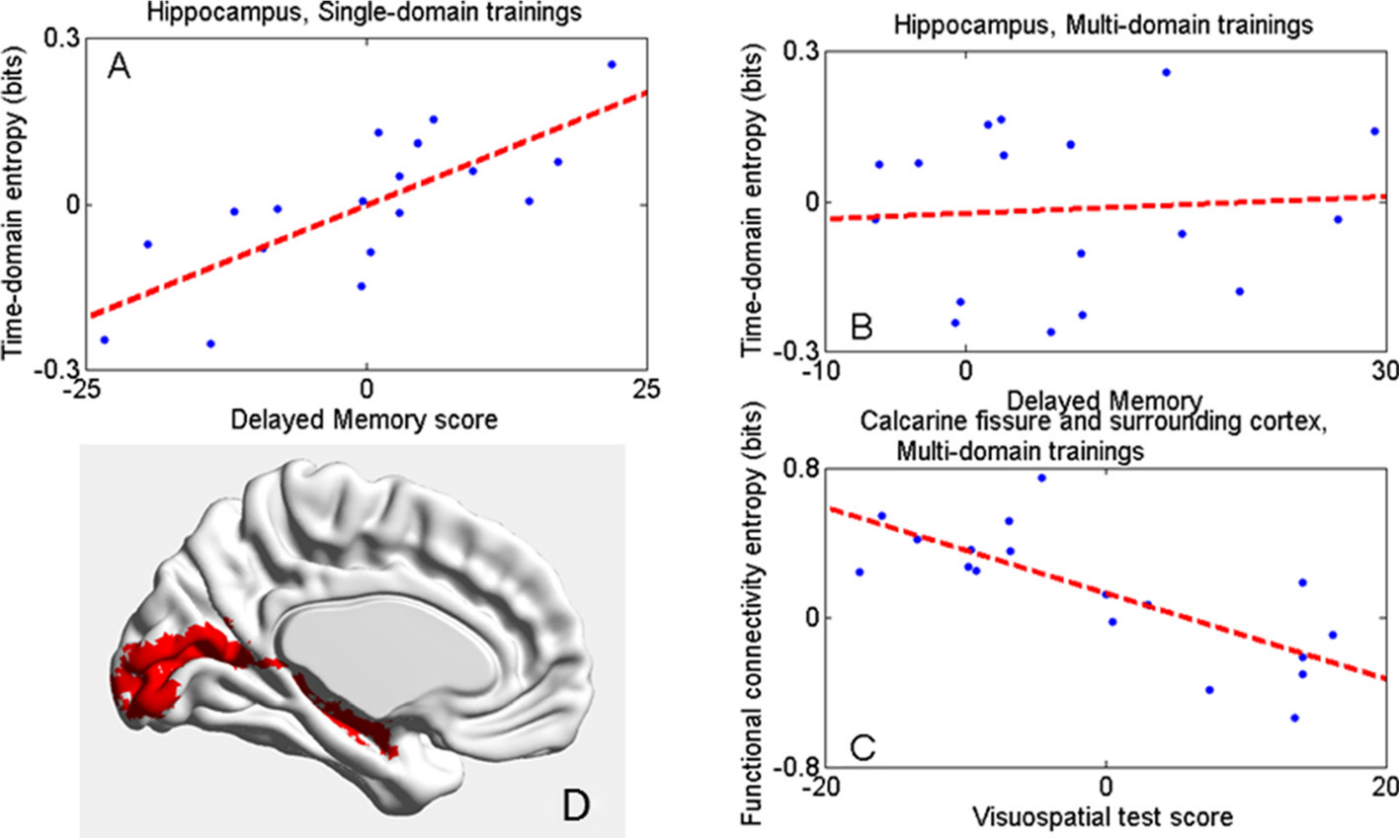
The correlation between cognitive capacity and resting state fMRI. Panel (**a**) and (**b**): Regional time-domain entropy for the hippocampus was significantly and positively correlated with delayed memory in the single-domain group (*r =* 0.760, *p =* 2.56 × 10^−4^) and positively (but not significant) correlated with delayed memory in the multi-domain group. Panel (**c**): In the multi-domain group, regional functional entropy of the calcarine fissure and the surrounding cortex was significantly negatively correlated with the visuospatial test (*r = −*0.758, *p =* 2.72 × 10^−4^). Panel (**d**): The regions referred to in panels (**a**) and (**b**) are shown

We found that the change of regional entropy in some regions correlated significantly with improvements in behavioral data. As shown in Fig. 6, regional time-domain entropy for the hippocampus was significantly and positively correlated with delayed memory in the single-domain group (*r =* 0.760, *p =* 2.56 × 10^−4^) and positively (but not significant) correlated with delayed memory in the multi-domain group. In the multi-domain group, regional functional entropy of the calcarine fissure and the surrounding cortex was significantly negatively correlated with the visuospatial test (*r = −*0.758, *p =* 2.72 × 10^−4^). Additionally, entropy trends in the two regions were significantly reduced in the training groups (*p* = 0.050).

## Discussion

In the results, we analyzed changes in the entropy of time series and functional connectivity from resting-state fMRI data. The trend of the functional entropy to increase, as observed in aging individuals, was significantly reduced in both groups that underwent cognitive training. Moreover, reduced lateralization, assessed by different entropy indices, was significantly suppressed in the multi-domain cognitive training group. Further, differences in gray matter changes were reduced in both training groups. Finally, behavioral improvements in memory and attention were significantly correlated with brain activity changes after training. In conclusion, our results provide explicit evidence that cognitive training can induce plastic changes at the level of intrinsic activity patterns in aging individuals. This is mainly through modifications in functional connectivity and brain structure, which are thus likely to be part of the neural mechanisms underlying the effects of cognitive training.

To our knowledge, this study is the first exploration of the effects of CogTr on functional connectivity in the aging brain from different aspects, as measured by entropy. Combining the analysis cognitive behavioral data, we conclude that while both types of CogTr could improve cognitive capacity in aging individuals, multi-domain training has more advantages in the cognitive domains that it affects.

We should emphasize that our results are primarily based on the informational concept of entropy, at the time and functional connectivity levels. To have a direct comparison with more conventional approaches, we analyzed the changes in functional connectivity between 4005 links in the different groups. We found one link that survived Bonferroni correction in the multi-domain training group, implying that connectivity was significantly improved with multi-domain training. The link in question was from the amygdala (right hemisphere) to the middle orbitofrontal cortex (right hemisphere). Li et al. explored the effects of multimodal intervention over a 6-week period, comprising cognitive training, Tai Chi exercise, and group counseling in the elderly, and found that intervention selectively affected resting-state functional connectivity between the medial prefrontal cortex and medial temporal lobe, which has some common features with our result. Differences could be because while they assessed short-term effects, we assessed long-term ones [50]. No links appeared to be correlated in the single-domain training group. Thus, consistent with the results from the entropy comparisons, multi-domain training is more effective than single-domain training. Perhaps it is not surprising that our entropy approach has revealed more information than the direct comparison using functional connectivity alone.

The results also suggest that the target of CogTr should be the dysfunction of cortical circuits that have been assumed to play a role in cognitive aging [51, 52]. The logic is given below. Extensive experimental data indicates that the number of neurons decreases significantly with age, as does the number of excitatory receptors (especially NMDA) [53]. Furthermore, changes in functional entropy with age can be simulated by computation models that include decreasing excitatory receptor number as a parameter [33]. CogTr may act to slow down the rate at which entropy increases with age by preventing the loss of the density of receptors on dendritic spines. This hypothesis could be tested in a diffusion-tensor imaging study.

At the regional level, training in the multi-domain group slowed down the decrease of time-domain entropy in the opercula part of the inferior frontal gyrus, the medial part of the superior frontal gyrus, and the thalamus. Similarly, training in the single-domain group significantly reduced the decrease in time-domain entropy in the cuneus. These results suggest that regions involved in speech production [54], self-awareness [55], relaying sensory and motor signals [56], and visual processing [57] are sensitive to CogTr. This study showed that in the multi-domain CogTr group, the paracentral lobule was where increasing functional entropy was significantly reduced. This is the medial portion of the superior frontal gyrus, continuous with the precentral and postcentral gyrus on the lateral surface. Salvador et al. found that functional connectivity for the paracentral lobule clustered statistically with resting-state activity of other frontal and parietal regions associated with spatial attention and motor functioning [58]. Therefore, improvement of functional connectivity in the paracentral lobule may be the neural basis for the improvement of attention capacity in the multi-domain CogTr group.

We also explored the effect of CogTr on the lateralization of time-domain and functional entropy. Lateralization can be viewed as a marker of brain activity efficiency. Consistent with a study having a larger dataset and wider age distribution [33], we found that functional entropy differences between the left and right hemisphere decreased with age. This decrease was significantly reduced in the multi-domain CogTr group, making the aging brain seem “younger”. Single-domain training did not have a similar result, suggesting that multi-domain CogTr could hold more advantages than single-domain CogTr. Multi-domain training might be enhanced by increasing the efficiency of multi-cognition domain-related regions and interactions between them.

At the regional level, the rate at which entropy asymmetry decreased was significantly reduced in the middle temporal gyrus, orbital part of superior frontal gyrus, and putamen of those in the multi-domain group. In fMRI experiments, these regions have been related to self-awareness [55], movement regulation [59], and several types of learning [60]. The results of the present study indicate that multi-domain training could increase the efficiency of those regions. Furthermore, we investigated gray matter hemisphere asymmetry using T1 image data. Consistent with studies of larger datasets with wider age distributions, the gray matter size difference between the left and right hemisphere significantly decreased with age in all three groups. However, the degree to which the asymmetry decreased was significantly less in the CogTr groups, thereby providing anatomical proof for the change in functional connectivity asymmetry.

To determine whether the entropy changes were relevant to behavior, we examined the relationship between the two. Changes in regional time-domain entropy of the hippocampus correlated significantly and positively with the change in delayed memory observed in the single-domain group, indicating the role of the hippocampus in the delayed memory process. In contrast, changes of regional functional entropy in the calcarine fissure and the surrounding cortex were significantly and negatively correlated with the change in visuospatial behavior observed in the multi-domain group. These regions function in primary sensory abilities such as vision and audition [61].

Several aspects of the present study should be taken into account when interpreting our results. First, the sample size was modest and may be susceptible to type II errors, obscuring true-positive effects. Bigger sample sizes are needed to verify the effect of CogTr on the aging brain. Second, the addition of a control group of young subjects could help determine whether CogTr training-induced changes are specific to older participants or whether they could also benefit younger adults. It would also help more directly determine whether the brains in the intervention groups get “younger” than those of the aging controls. Finally, it should be again emphasized that most of our results arise from an analysis based on the notion of entropy. We found that in the multi-domain training group, there was one functional link that was significantly modified, but there was no such link in the single-domain training group. In this sense, multi-domain training was better than single-domain training, and was consistent with the results based on entropy. Therefore, the entropy analysis revealed reliable results. We note that our entropy approach revealed more information than a direct comparison using functional connectivity analysis alone, which implies that entropy had more statistical power. We also conducted a functional connectivity analysis and found results similar to the entropy experiments (Additional file 1, Section 8). This could be attributed to global aspects of entropy. This is the reason entropy was selected to measure resting-state functional MRI.

## Conclusion

In conclusion, CogTr can induce plastic changes at the level of intrinsic activity patterns in older people, mainly by modifying functional connectivity. Thus, changes in functional connectivity are likely to be a key neural mechanism underlying the CogTr effect. Multi-domain CogTr demonstrated more advantages than single-domain CogTr, perhaps because multi-domain CogTr involves more regions related to cognition. Larger sample sizes are urgently needed to study CogTr-induced changes in brain activity, cognition capacity, and their relationship.

## Additional file

Additional file 1: **Cognitive training can reduce the rate of cognitive aging: a neuroimaging cohort study.** (DOCX 443 kb)

## Abbreviations

CogTr: Cognitive training
BOLD: Blood oxygenation level-dependent signals
fMRI: Functional magnetic resonance imaging
ROI: Region of interest
