# Supplementary material for: Cognitive training can reduce the rate of cognitive aging: a neuroimaging cohort study
Source: BMC Geriatr. 2016 Jan 14;16:12. doi: 10.1186/s12877-016-0194-5 (PMC4712458; doi:10.1186/s12877-016-0194-5)
Supplement: Additional file 1: — Cognitive training can reduce the rate of cognitive aging: a neuroimaging cohort study. (DOCX 443 kb) [file 12877_2016_194_MOESM1_ESM.docx]

**Additional file 1**

Cognitive Training Can Reduce the Rate of Cognitive Aging

- Evidence from Cohort Neuroimaging Data

Ting Li^1,*^, Ye Yao^2,6,*^,Yan Cheng^3,*^,Bing Xu^2,6^, Xinyi Cao^1^, David Waxman^2^, Wei Feng^3^, Yuan Shen^4^, Qingwei Li^3^, Jijun Wang^1^, Wenyuan Wu^3^, Chunbo Li^1,+^, Jianfeng Feng^2,5,6,+^

^1^ Shanghai Key Laboratory of Psychotic Disorders, Shanghai Mental Health Center, Shanghai Jiao Tong University School of Medicine, Shanghai, China

^2^ Centre for Computational Systems Biology, Fudan University, Shanghai, China

^3^ Department of Psychiatry, Tongji Hospital of Tongji University, Shanghai, China

^4^ Department of Psychiatry, Tenth People's Hospital of Tongji University, Shanghai, China

^5^ Shanghai Center for Mathematical Sciences, Fudan University, Shanghai, China

^6^ Department of Computer Science, University of Warwick, Coventry CV4 7AL, UK

*: Equal contributors.

+: Equal contributors.

Ting Li^1,*^

Email: liting2319@163.com

Ye Yao^2,6,*^

Email: yyao@fudan.edu.cn

Yan Cheng^3,*^

Email: xiaoyanzi2001505@163.com

Bing Xu^2,6^

Email: bingxu1005@gmail.com

Xinyi Cao^1^

Email: platoreki@gmail.com

David Waxman^2^

Email: dwaxman.biology@gmail.com

Wei Feng^3^

Email: ffww06@163.com

Yuan Shen^4^

Email: kmsharonshen@gmail.com

Qingwei Li^3^

Email: lianocd@tongji.edu.cn

Jijun Wang^1^

Email: jijunwang27@163.com

Wenyuan Wu^3^

Email: wuwy@tongji.edu.cn

Chunbo Li^1,+^

^+^ Corresponding author

Email: chunbo_li@163.com

Jianfeng Feng^2,5,6,+^

^+^ Corresponding author

Email: jffeng@fudan.edu.cn

Table of Contents

[1 Names and abbreviations of AAL brain regions 4](#_Toc435451683)

[2 Methods about T1-image data 5](#_Toc435451684)

[2.1 Subjects 5](#_Toc435451685)

[2.2 Data Preprocessing 5](#_Toc435451686)

[2.3 Gray matter hemisphere asymmetry change with age 6](#_Toc435451687)

[3 Methods about time-domain entropy 7](#_Toc435451688)

[3.1 Subjects 7](#_Toc435451689)

[3.2 Data Preprocessing 7](#_Toc435451690)

[3.3 Approximate Entropy 8](#_Toc435451691)

[3.4 Time-domain Entropy Calculation 8](#_Toc435451692)

[4 Cognitive training reduces the gray matter asymmetry decrease rate with age 11](#_Toc435451693)

[4.1 Methods 11](#_Toc435451694)

[4.2 Results 11](#_Toc435451695)

[5 Time-domain entropy decreases with age 13](#_Toc435451696)

[6 Functional connectivity entropy difference between the left and right hemisphere decreases with age 14](#_Toc435451697)

[7 Regional entropy asymmetries change with age 15](#_Toc435451698)

[8 Functional Connectivity Analysis 16](#_Toc435451699)

[9 References 18](#_Toc435451700)

# 1 Names and abbreviations of AAL brain regions

**Names and abbreviations of Regions Of Interest (ROIs)**.

| **Region** | **Abbr.** | **Region** | **Abbr.** |
| --- | --- | --- | --- |
| Amygdala | AMYG | Orbitofrontal cortex (middle) | ORBmid |
| Angular gyrus | ANG | Orbitofrontal cortex (superior) | ORBsup |
| Anterior cingulate gyrus | ACG | Pallidum | PAL |
| Calcarine cortex | CAL | Paracentral lobule | PCL |
| Caudate | CAU | Parahippocampal gyrus | PHG |
| Cuneus | CUN | Postcentral gyrus | PoCG |
| Fusiform gyrus | FFG | Posterior cingulate gyrus | PCG |
| Heschl gyrus | HES | Precentral gyrus | PreCG |
| Hippocampus | HIP | Precuneus | PCUN |
| Inferior occipital gyrus | IOG | Putamen | PUT |
| Inferior frontal gyrus (opercula) | IFGoperc | Rectus gyrus | REC |
| Inferior frontal gyrus (triangular) | IFGtriang | Rolandic operculum | ROL |
| Inferior parietal lobule | IPL | Superior occipital gyrus | SOG |
| Inferior temporal gyrus | ITG | Superior frontal gyrus (dorsal) | SFGdor |
| Insula | INS | Superior frontal gyrus (medial) | SFGmed |
| Lingual gyrus | LING | Superior parietal gyrus | SPG |
| Middle cingulate gyrus | MCG | Superior temporal gyrus | STG |
| Middle occipital gyrus | MOG | Supplementary motor area | SMA |
| Middle frontal gyrus | MFG | Supramarginal gyrus | SMG |
| Middle temporal gyrus | MTG | Temporal pole (middle) | TPOmid |
| Olfactory | OLF | Temporal pole (superior) | TPOsup |
| Orbitofrontal cortex (inferior) | ORBinf | Thalamus | THA |
| Orbitofrontal cortex (medial) | ORBmed |  |  |

Table S1. Names and abbreviations of AAL brain regions

# 2 Methods about T1-image data

## 2.1 Subjects

The T1-image dataset contained four groups of samples from Kunming, Nanjing, Shanghai and Taipei. There were 496 samples at all, 252 males and 244 females, ranging from 19 to 87 years old, with a mean age of 45.1 ± 20.0 years.

The Kunming dataset came from Kunming Institute of Zoology, Chinese Academy of Sciences, 111 males and 188 females, with a mean age of 35.4 ± 12.6 years. The Nanjing dataset came from Department of Medical Imaging, Jinling Hospital, Nanjing University School of Medicine, 8 males and 12 females, with a mean age of 22.7 ± 1.5 years. The Shanghai dataset came from Prof. Chunbo Li’s lab, who was one of the authors of the paper. 73 of them were males and 44 were females. They held a mean age of 70.4 ± 3.5 years. The last, Taipei dataset came from Department of Biomedical Imaging and Radiological Sciences, National Yang-Ming University. All of them were males with a mean age of 51.3 ± 21.5 years.

## 2.2 Data Preprocessing

Voxel-based morphometry [[1](#_ENREF_1)] (VBM) was used to provide a voxel-wise assessment of volumetric differences. All preprocessings were performed by SPM8 [[2](#_ENREF_2)]. High resolution T1 images were first segmented into gray matter, white matter and cerebrospinal fluid in the native space by applying VBM8. The gray matter and white matter images were then iteratively aligned to an increasingly crisp average template by DARTEL. After that, we calculated the probability of every voxel which it would be gray matter or white matter. The probability times the voxel volume could be considered as the size of gray matter and white matter in every voxel. Finally, each T1 image was normalized to standard MNI space [[3](#_ENREF_3)] and an inverse deformation field from template to image would be gotten. We transferred the template in MNI space to the native space of every sample and counted the gray matter and white matter volumes of the regions of interest (ROIs) based on AAL atlas [[3](#_ENREF_3)]. The ROIs are listed in Table S1.

## 2.3 Gray matter hemisphere asymmetry change with age

After data preprocessing, we summed the gray matter size based on the AAL atlas, separately in the left and right hemisphere. Then we calculated the difference of the gray matter volume between the left and right hemisphere. As shown in Figure S1, the difference significantly decreased with age (*r =* -0.205, *p* = 4.30 × 10^-6^). If we removed the effect of gender difference by partial correlation coefficient [[4](#_ENREF_4)], the relationship between the gray matter volume difference with age is still significant (*r =* -0.156, *p* = 4.79 × 10^-4^). In addition, this implied that the gray matter in the left hemisphere atrophied faster than that in the right hemisphere.


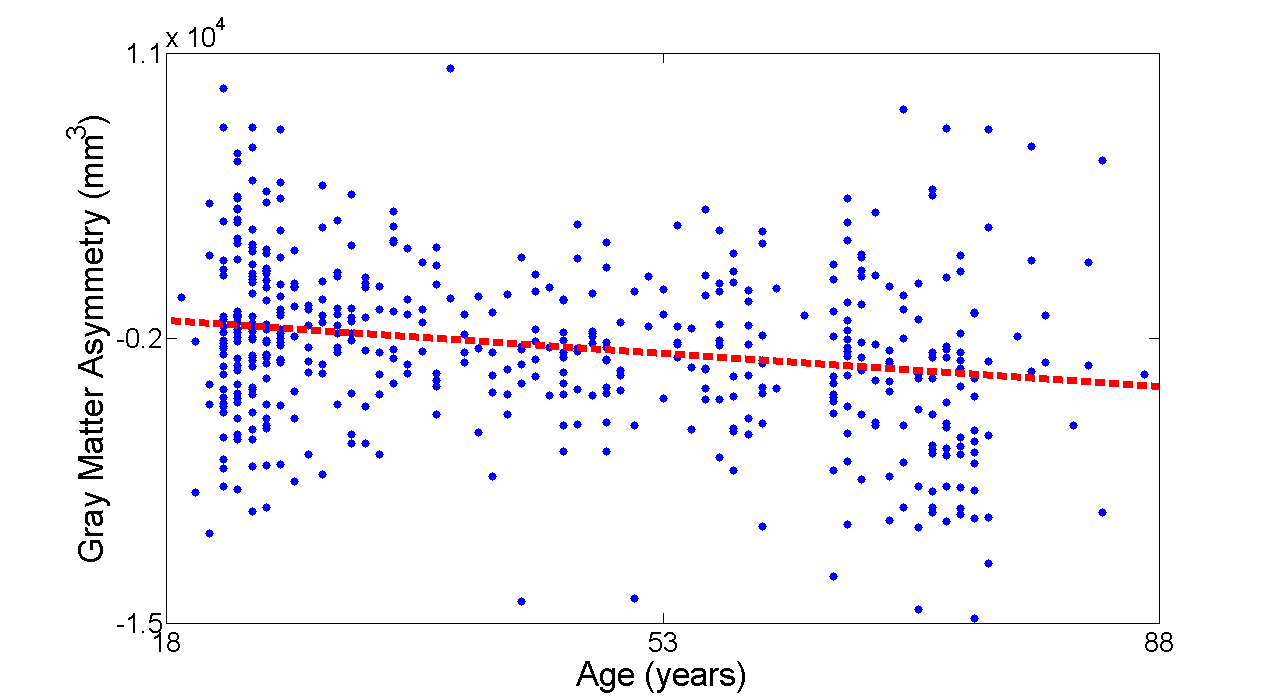


Figure S1. Gray matter asymmetry decreases with age

The difference of the gray matter volume between the left and right hemisphere significantly decreased with age (r = -0.205, *p* = 4.30 × 10^-6^).

# 3 Methods about time-domain entropy

## 3.1 Subjects

Our resting state BOLD signal meta-analysis about time-domain entropy included 20 datasets, with a total of 755 samples. These covered a range of individuals from 18 to 76 years of age. 380 of them are males and 375 samples are females. They hold a mean age of 36.3 ± 19.0 years. We excluded samples of poor quality.

Note that 18 of these datasets came from the 1000 Functional Connectomes Project (<http://fcon_1000.projects.nitrc.org/>), where data came from all over the world including China, Britain and United States. In these 18 data sets, there were 590 samples, with ages ranging from 18 to 73 years, with 259 of them male. The mean age was 29.0 ± 12.1 years. Details are listed in Table S2.

There was also one dataset of elderly people, covering 117 samples from one of the authors of this paper, Prof. Chunbo Li’s lab, the Department of Psychiatry, Tongji Hospital, Tongji University School of Medicine and Department of Biological Psychiatry, Shanghai Mental Health Centre, Shanghai Jiao Tong University School of Medicine. In this dataset, 73 were male. The mean age was 70.42 ± 3.52 years. With a designed health status checklist, we excluded individuals with: obvious cognitive decline, a diagnosis of AD, serious functional decline (having difficulty with independent living), as well as individuals with major medical or psychiatric conditions such as cancer, current chemotherapy/radiation treatment, major depression, and schizophrenia.

The last dataset was from the Department of Biomedical Imaging and Radiological Sciences, National Yang-Ming University, Taipei, Taiwan and the Brain Connectivity Laboratory, Institute of Neuroscience, National Yang-Ming University, Taipei, Taiwan. There were 48 samples in the dataset. All were male and covered a range of ages from 21 to 76 years. The mean age was 43.8 ± 17.0 years and all individuals were normal and healthy.

## 3.2 Data Preprocessing

The data preprocessing is the same as that in the Methods section in the main text.

## 3.3 Approximate Entropy

In 1981, Dr. Shaw recognized that a measure of the rate of information generation of a chaotic system is a useful parameter [[5](#_ENREF_5)]. Two years later, Dr. Grassberger and Dr. Procaccia [[6](#_ENREF_6)] developed a formula, motivated by the K-S (Kolmogorov-Sinai) entropy [[7](#_ENREF_7)], to calculate such a rate from time series data. After another two years, Dr. Eckmann and Dr. Ruelle [[8](#_ENREF_8)] improved the formula to calculate the K-S entropy for the physical invariant measure presumed to underlie the data distribution, which was named E-R Entropy [[9](#_ENREF_9)]. These formulas have become the standard entropy measures for use with time- series data [[10](#_ENREF_10)].

We next indicated the E-R (Eckmann-Ruelle) entropy [[8](#_ENREF_8)] formula. Given a time-series of data, $u\left( 1 \right), u\left( 2 \right),\ldots,u\left( N \right)$, from measurements equally spaced in time and a positive integer, $m$, form a sequence of vectors, $x\left( l \right), x\left( 2 \right), \ldots, x(N-m+1)$ in $R^{m}$, defined by $x(i)=[u(i), u\left( i+1 \right) , \ldots, u(i+m-1)]$. Next, fix a positive real number, $r$, and define for each $i$, $1\leq i\leq N - m + 1$,

$C_{i}^{m}(r)=(number of j such that d\left[ x\left( i \right), x\left( j \right) \right]\leq r)/(N-m+1)$, (1)

where,

$d[x(i), x(j)]=\max_{k=1,2, \ldots, m}(\left| u\left( i+k-1 \right)-u(j+k-1) \right|)$. (2)

Then, define

$\phi^{m}\left( r \right)= \left( N-m+1 \right)^{-1}\sum_{i=1}^{N-m+1} \log C_{i}^{m}\left( r \right)$. (3)

And,

$E-R entropy = \lim_{r\to0}\lim_{m\to+\infty}\lim_{N\to+\infty}[\phi^{m}\left( r \right)-\phi^{m+1}\left( r \right)]$. (4)

As shown in Dr. Pincus’s work [[10](#_ENREF_10)], the approximate entropy (denoted ApEn) was calculated by implementing the formula in Eq. (4) and defining the statistic. Given $N$ data points,

$ApEn(m, r, N)=\phi^{m}\left( r \right)-\phi^{m+1}\left( r \right)$. (5)

Moreover, as shown in Dr. Pincus’s work [[10](#_ENREF_10)] (Theorem 3), the approximate entropy in some case was one kind of expressions of Shannon entropy.

In the first-order stationary Markov chain (discrete state space $X$) case, with $r<min(\left| x-y \right|,x\neq y, x$ and $y$ state space values$)$, a.s. for any $m$,

$ApEn(m, r)=-\sum_{x\in X} \sum_{y\in X} \pi(x)p_{\mathrm{xy}} log(p_{\mathrm{xy}})$. (6)

## 3.4 Time-domain Entropy Calculation

After data preprocessing, the time series were extracted in each ROI by averaging the signals of all voxels within the region. The 90 regions were based on a selected atlas, say the AAL Template [[3](#_ENREF_3)].

Then we calculated Approximate Entropy [[10](#_ENREF_10)] of every time series with the window length m = 1 and the regularity *r =* 0.12 × SD (Standard Deviation) of the time series. We called this regional time-domain entropy of every single brain region. Afterwards, we averaged the regional time-domain entropy of all the brain regions and considered this as the approximate entropy of the whole brain. This was because that all the approximate entropy of different brain regions can be considered as a realization of a random variable, which can be approached by averaging. We named this to be time-domain entropy.

It should be emphasized that the ratio between r and SD is selected by realistic data. We calculated and averaged the time domain entropy of the samples from Taiwan with different regularity r. As shown in Figure S2, the time-domain entropy hit the maximum when *r =* 0.12 × SD. That’s why we selected the ratio to be 0.12.

Moreover, the base of the logarithm in the paper is 2. Thus, the unit of the entropy in the paper is the bit (binary digit).

| Database | Quantity | Male / Female | Age (years) |
| --- | --- | --- | --- |
| Atlanta | 27 | 12 / 15 | 29.9 ± 8.6 |
| Baltimore | 23 | 8 / 15 | 29.3 ± 5.5 |
| Beijing_Zang | 193 | 75 / 118 | 21.2 ± 1.8 |
| Berlin_Margulies | 23 | 12 / 11 | 30.1 ± 5.1 |
| Dallas | 24 | 12 / 12 | 42.6 ± 20.1 |
| ICBM | 36 | 15 / 21 | 38.2 ± 17.6 |
| Leiden_2180 | 10 | 10 / 0 | 23.4 ± 2.5 |
| Leiden_2200 | 19 | 11 / 8 | 21.7 ± 2.6 |
| Leipzig | 37 | 16 / 21 | 26.2 ± 5.0 |
| Milwaukee_b | 46 | 15 / 31 | 53.5 ± 5.8 |
| NewHeaven_b | 16 | 8 / 8 | 26.9 ± 6.3 |
| Newark | 18 | 9 / 9 | 24.3 ± 4.0 |
| Orangeburg | 20 | 15 / 5 | 40.7 ± 11.0 |
| Oulu | 22 | 7 / 15 | 21.3 ± 0.6 |
| PaloAlto | 17 | 2 / 15 | 32.5 ± 8.1 |
| Pittsburgh | 14 | 8 / 6 | 36.0 ± 8.6 |
| Queensland | 18 | 11 / 7 | 26.3 ± 3.7 |
| Saintlouis | 27 | 13 / 14 | 25.3 ± 2.3 |
| Whole Database | 590 | 259 / 231 | 29.0 ± 12.1 |

Table S2. Detailed information of 18 databases from the FCON 1000 Project


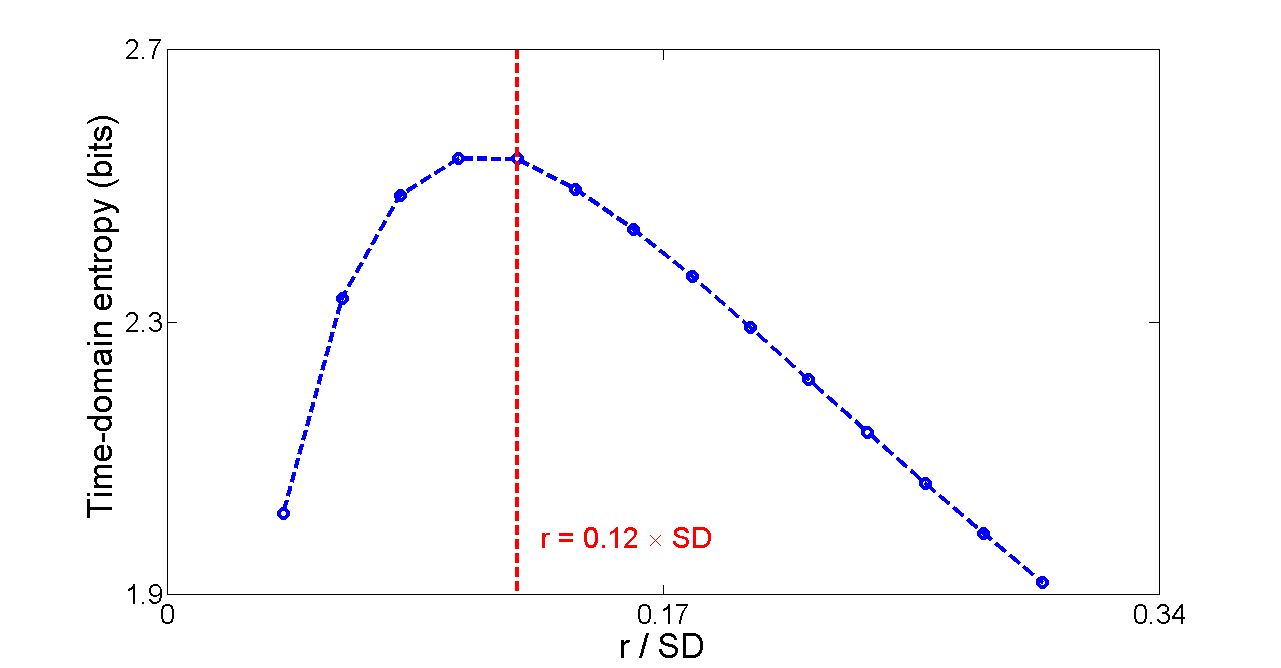


Figure S2. Time-domain entropy versus regularity / standard deviation

The time-domain entropy hit the maximum when *r =* 0.12 × SD (Standard Deviation).

# 4 Cognitive training reduces the gray matter asymmetry decrease rate with age

## 4.1 Methods

**Subjects**

The subjects we used here were the same as that in the Methods section in the main text.

**Data Acquisition**

High-resolution T1-weighted anatomical images were acquired in the sagittal orientation using a magnetization-prepared rapid gradient-echo sequence (repetition time = 1900 ms, echo time = 3.43 ms, flip angle = 9, field of view = 256 × 256 mm^2^, matrix size = 256 × 256, slice thickness = 1 mm, voxel size = 0.9375 × 0.9375 × 1 mm^3^ and 160 slices) on each subject.

**Data Preprocessing**

The data preprocessing steps are the same as that in Supplementary Materials Section 2.2.

## 4.2 Results

As shown in Figure S1, The difference of the gray matter volume between the left and right hemisphere significantly decreased with age. Here, we demonstrated that the gray matter asymmetry decrease rate was reduced by cognitive training.

Firstly, we calculated the gray matter asymmetry change of all the samples in one year. After that, we found the relationship between the asymmetry change with the age, sex and education in the controls by linear regression [[11](#_ENREF_11)]. Then, we applied the controls as the baseline and predicted the asymmetry decrease in the multi-domain and single-domain training group with their age, sex and education. Therefore, we can compare with the difference between the predictions with the actual ones that we extracted from the data, which can be considered as the effect of the cognitive training.

As shown in Figure S3, the gray matter asymmetry decrease rate was significantly reduced in the multi-domain training (t-test = 2.93, *p =* 0.005) and single-domain training group (t-test = 3.33, *p =* 0.002). This can be considered as another evidence that cognitive trainings did good to our brains.


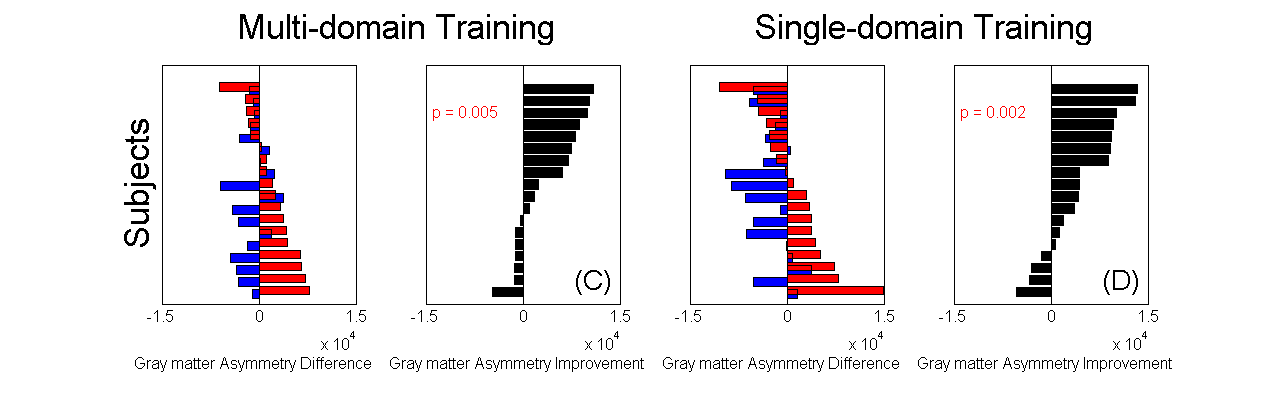


Figure S3. Cognitive training reduces the gray matter asymmetry decrease rate

The gray matter asymmetry differences (black nodes) between predictions (blue nodes) with actual ones (red nodes) indicated that the gray matter asymmetry decrease rate was significantly reduced in multi-domain training (t test = 2.93, *p =* 0.005) and single-domain training group (t test = 3.33, *p =* 0.002). The rank of the subjects is arranged by the gray matter asymmetry volume.

# 5 Time-domain entropy decreases with age

We calculated the trend of time-domain entropy with age by the database described in Supplementary Materials Section 3. Thus, we found that time-domain entropy significantly (*r =* -0.419, p < 1 × 10^-322^) decreased with age. As shown in Figure S4, a mean rate of decrease of the entropy of 0.0027 bits/year was found. Moreover, males held higher entropy than females. In addition, the linear correlation between entropy and age was strongly significant (*r =* -0.385, *p =* 7.66 x 10^-15^ for males and *r =* -0.455, p < 1 × 10^-322^ for females).


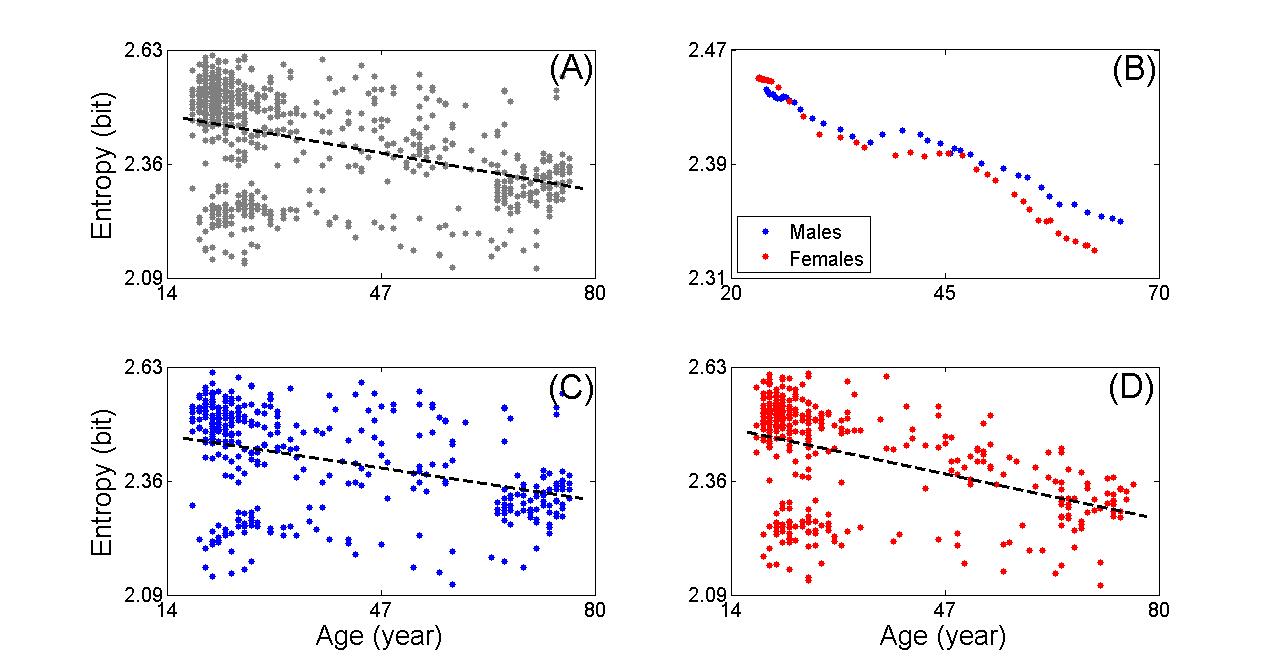


Figure S4. Time-domain entropy decreased with age

Panel (A) is a plot of the entropy of individuals versus their age (pooling results from males and females). A mean rate of decrease of the entropy of 0.0027 bits/year was found from the data. Panel (b) contains a plot of the running average of the entropy, versus age, with a window of width of 30 years adopted. Males held higher entropy than females. Panels (C) and (D) plot the entropy versus age of males and females. The linear correlation between entropy and age is strongly significant (*p =* 7.66 x 10^-15^ for males and p < 1 × 10^-322^ for females)

In addition, we certificated that all the regional time-domain entropy significantly decreased with age.

# 6 Functional connectivity entropy difference between the left and right hemisphere decreases with age

With the database used in the paper, ‘The Increase of the Entropy of the Human Brain with Age’, in press, we calculated the functional connectivity entropy difference between the left and right hemisphere. As shown in Figure S5, the entropy difference significantly (*r =* -0.065, *p =* 0.021) decreased with age.


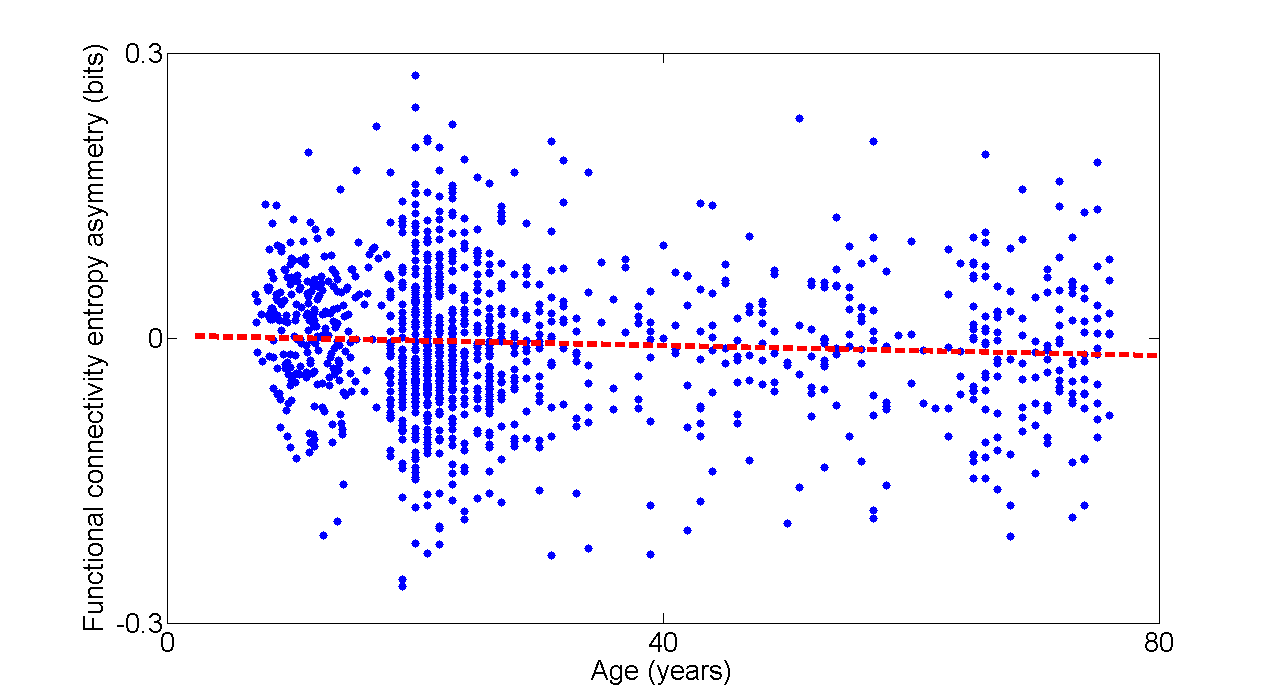


Figure S5. Functional connectivity entropy asymmetry decreases with age

The functional connectivity entropy difference between the left and right hemisphere significantly decreased with age (r = -0.065, *p =* 0.021).

# 7 Regional entropy asymmetries change with age

Additionally, we calculated the entropy asymmetry change rate with age in regional level and found out that time-domain entropy in middle temporal gyrus significantly (*r =* 0.082, *p =* 0.024) increased with age; functional connectivity entropy in the orbital part of superior frontal gyrus significantly (*r =* -0.153, *p =* 5.13 × 10^-8^) decreased with age and that in putamen significantly (*r =* 0.109, *p =* 2.20 × 10^-4^) increased with age.


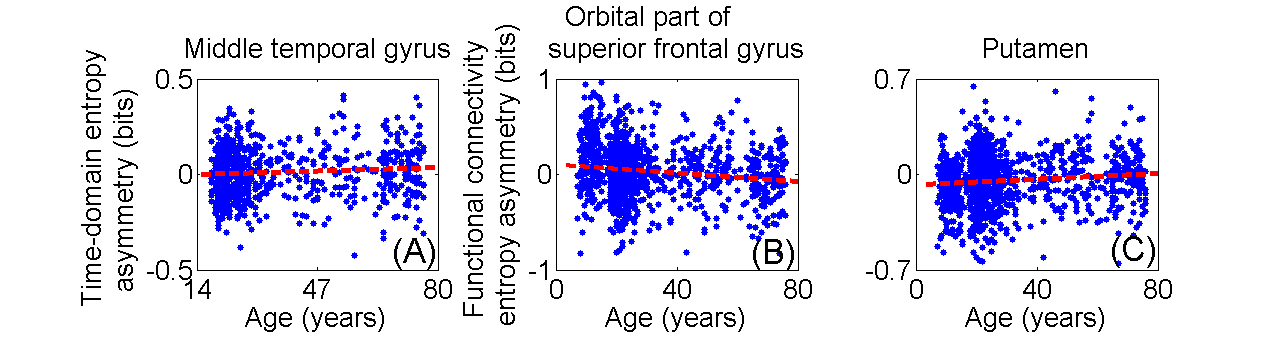


Figure S6. Regional entropy asymmetries change with age

Time-domain entropy in middle temporal gyrus significantly (*r =* 0.082, *p =* 0.024) increased with age; functional connectivity entropy in the orbital part of superior frontal gyrus significantly (*r =* -0.153, *p =* 5.13 × 10-8) decreased with age and that in putamen significantly (*r =* 0.109, *p =* 2.20 × 10-4) increased with age.

# 8 Functional Connectivity Analysis

It should be emphasized that our results are mainly based on an informational concept, namely entropy, at the time and functional connectivity levels. To have a direct comparison with more conventional approaches, we have analyzed the change of the functional connectivity of the 4005 links in the different groups. We found that in the multi-domain training group, there is one link which can survive the Bonferroni correction (p = 7.2 × 10-6 < 0.05 / 4005) as shown in Figure S7. This implied the link was significantly improved with Multi-domain training. This is the link from the amygdala (right hemisphere) to the middle orbitofrontal cortex (right hemisphere). There are no links in the single-domain training group that can pass the Bonferroni correction. Therefore, the multi-domain training was, by this objective criterion, better than the single-domain training, and consistent with the results from entropy. This is, perhaps, not surprising: our entropy approach has revealed more information than a direct comparison using functional connectivity alone.


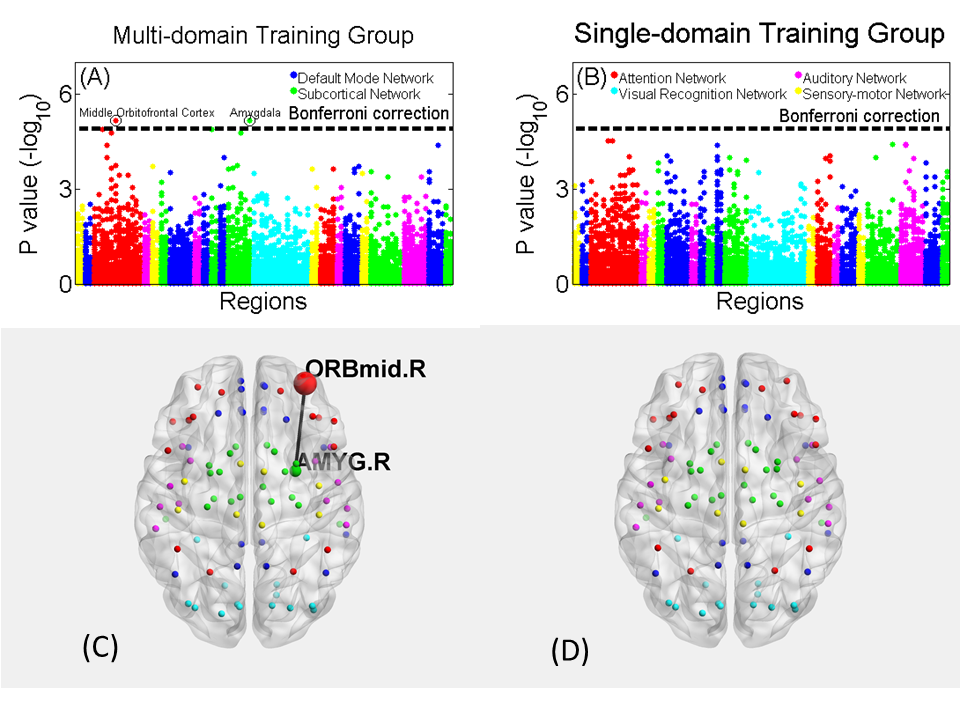


Figure S7. Functional Connectivity Change in training groups

Panels (A) & (C) In the Multi-domain training group, the p value of the link from Amygdala (Right hemisphere) to Middle Orbitofrontal Cortex (Right hemisphere) can pass the Bonferroni correction ( p = 7.2 × 10-6 < 0.05 / 4005).

Panels (B) & (D) In the single-domain training group, there are no links whose p value can pass the Bonferroni correction.

# 9 References

1. Ashburner J and Friston KJ. Voxel-based morphometry—the methods*.* Neuroimage. 2000; 11(6): 805-21.

2. Frackowiak RS, *Human brain function*. 2004: Academic Press.

3. Tzourio-Mazoyer N, Landeau B, Papathanassiou D, Crivello F, Etard O, Delcroix N, et al. Automated anatomical labeling of activations in SPM using a macroscopic anatomical parcellation of the MNI MRI single-subject brain*.* Neuroimage. 2002; 15(1): 273-89.

4. Kleinbaum DG, Kupper LL, Muller KE, and Nizam A, *Applied regression analysis and multivariable methods*. 1998: Duxbury Press 3rd Ed. Pacific Grove.

5. Shaw R. Strange Attractors, Chaotic Behaviour and Information Flow*.* Zeitschrift für Naturforschung. 1981; 36(A): 80-112.

6. Grassberger P and Procaccia I. Estimation of the Kolmogorov entropy from a chaotic signal*.* Physical review A. 1983; 28(4): 2591-3.

7. Downarowicz T, *Entropy in dynamical systems*. Vol. 18. 2011: Cambridge University Press.

8. Eckmann J-P and Ruelle D. Ergodic theory of chaos and strange attractors*.* Reviews of modern physics. 1985; 57(3): 617.

9. Bunimovich L, Dani S, Dobrushin R, Jakobson M, Kornfeld I, Maslova N, et al., *Dynamical systems, ergodic theory and applications*. Vol. 1. 2000: Springer.

10. Pincus SM. Approximate entropy as a measure of system complexity*.* Proceedings of the National Academy of Sciences. 1991; 88(6): 2297-301.

11. Seber GA and Lee AJ, *Linear regression analysis*. Vol. 936. 2012: Wiley.
